# Prognostic significance of PD-L1 expression and ^18^F-FDG PET/CT in surgical pulmonary squamous cell carcinoma

**DOI:** 10.18632/oncotarget.18257

**Published:** 2017-05-29

**Authors:** Minghui Zhang, Dalong Wang, Qi Sun, Haihong Pu, Yan Wang, Shu Zhao, Yan Wang, Qiangyuan Zhang

**Affiliations:** ^1^ Department of Medical Oncology, Harbin Medical University Cancer Hospital, Harbin 150081, China; ^2^ Department of Radiology, The Second Affiliated Hospital of Harbin Medical University, Harbin 150040, China; ^3^ Department of PET/CT, Harbin Medical University Cancer Hospital, Harbin 150081, China; ^4^ Department of Radiology, Harbin Medical University Cancer Hospital, Harbin 150081, China; ^5^ Department of Medical Oncology, Heilongjiang Provincial Hospital, Harbin 150000, China

**Keywords:** programmed cell death ligand 1, squamous cell carcinoma, prognostic factor, PET-CT

## Abstract

Programmed cell death-ligand 1 (PD-L1) expression is commonly observed in non-small cell lung cancer (NSCLC). The prognostic value of PD-L1 expression and the maximum standardized uptake value (SUVmax) on 18F-Fluorodeoxyglucose positron emission tomography (^18^FDG-PET) in surgical pulmonary squamous cell carcinoma(SCC)remains unclear. Furthermore, the correlation between the SUVmax and PD-L1 expression has not been assessed. Thus, the purpose of this study was to investigate the correlation between PD-L1 expression and the SUVmax on ^18^FDG-PET and to examine the prognostic significance of PD-L1 expression and the SUVmax in surgical pulmonary SCC. Expression of PD-L1 was examined in 84 patients with resected SCC using immunohistochemistry. Positive PD-L1 expression in tumour cells was observed in 58.3% (49/84) of patients with SCC. High PD-L1 expression levels were significantly correlated with histological differentiation (P=0.006), and a high SUVmax was associated with histological differentiation (P=0.037), and lymph node metastasis (P=0.025). Spearman's test showed that there was a significant correlation between PD-L1 expression levels and the SUVmax. High PD-L1 expression levels and a high SUVmax were both independent risks factors for poor overall survival. Our results suggested that high PD-L1 expression levels and a high SUVmax was associated with poor prognosis in surgical pulmonary SCC. The existence of a statistically significant correlation between the SUVmax and PD-L1 expression levels justifies exploring the usefulness of the SUVmax as a predictor of PD-1/PD-L1 inhibitor activity.

## INTRODUCTION

Lung cancer, which can be broadly divided into small cell lung cancer (SCLC) and non-small cell lung cancer (NSCLC), is the most prevalent cancer worldwide [1]. Certain prognostic factors including advanced stage disease at diagnosis, poorly differentiated, abnormal activation of oncogene are predictive of poor survival in patients with NSCLC. Squamous cell carcinoma (SCC) accounts for approximately 30% of all cases of lung cancer [2]. The number of treatment options for lung adenocarcinoma (ADC) has increased in recent decades following the identification of sensitizing epidermal growth factor receptor (EGFR) mutations and anaplastic lymphoma kinase (ALK) rearrangements as molecular targets for effective agents [3]. However, most of the molecular target therapies used for the treatment of ADC are not indicated for SCC because the latter disease lacks specific genetic alterations. Blocking immunecheckpoints with monoclonal antibodies has emerged as a new therapeutic strategy for treating SCC and has yielded a favourable clinical outcome in affected patients [4]. Such results highlight the importance of immune checkpoints in SCC tumourigenesis.

The programmed death 1 (PD-1) pathway is a major immune checkpoint. The binding of PD-1 ligand (PD-L1) to PD-1 induces activated T-cell apoptosis or exhaustion, and agents that block this inhibitory signal have been shown to enhance T-cells activity [5]. Previous studies have shown that increased PD-L1 expression occurs in many solid tumours, including breast cancer [6], NSCLC [7], hepatocellular carcinoma[8], gastric cancer[9], colorectal cancer[10], renal cell carcinoma[11], and testicular cancer[12], as well as papillary thyroid cancer[13]. Several meta-analyses have demonstrated that high PD-L1 expression levels are correlated with adverse clinical and pathologic features, as well as an increased risk of death, in many cancer types [14–17]. However, the data regarding the prevalence and prognostic role of PD-L1 expression in SCC remain controversial. A meta-analyse demonstrated that NSCLC patients with high PD-L1 expression had a poor OS [18]. However, another meta-analysis did not indicate PD-L1 as a prognostic predictor for NSCLC [19].

18F-Fluorodeoxyglucose positron emission tomography (^18^FDG-PET) is an important noninvasive tool used for the diagnosing NSCLC and performing TNM staging in patients with the disease. The maximum standardized uptake value (SUVmax) on FDG-PET is widely used in clinical practice because of its simplicity [20]. A high SUVmax has been reported to be an important prognostic factor in patients with NSCLC [21]. Several studies have demonstrated the existence of a correlation between the uptake of FDG and the levels of biomarkers such as EGFR mutations [22], VEGF[23], Ki-67 [24] and COX2 [25]. However, the correlation between FDG uptake and PD-L1 expression has not been assessed.

Therefore, we conducted this study to investigate the correlation between PD-L1 expression and the SUVmax on FDG-PET and to examine the prognostic significance of PD-L1 expression and SUVmax in surgical SCC.

## RESULTS

### Patient demographics

**Figure 1:**
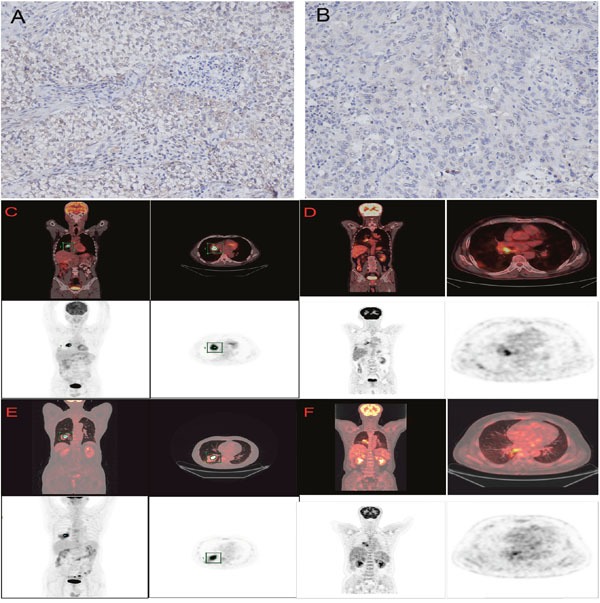
IHC staining for PD-L1 in pulmonary SCC patients **(A)** Positive PD-L1 expression. **(B)** Negative PD-L1 expression. **(C)**18F-FDG PET/CT in patients with pulmonary SCC. 71-year-old man with positive PD-L1 expression and a high SUVmax (13.3). **(D)** 57-year-old man with positive PD-L1 expression and a low SUVmax (6.9). **(E)** 65-year-old man with negative PD-L1 expression and a high SUVmax(19.1). **(F)** 59-year-old man with negative PD-L1 expression and a low SUVmax(6.8).

The clinical characteristics of the 84 patients enrolled in the study are presented in Table 1. The median age of the study population at the time of diagnosis was 63 years (range, 34–85 years), and the majority of patients (77.4%) were male. Sixty-one (72.6%) of patients were smokers, and 75% of the cohort comprised patients with stage I and II disease. After curative resection, 54 patients received platinum-based adjuvant chemotherapy, and 77.8% of patients were treated with up to 4 cycles of chemotherapy. A total of 12 patients (14.3%) received adjuvant chemotherapy followed by radiotherapy. The median follow-up time of was 27.8 months (range, 4.3-78 months). The median SUVmax was 11.2 (range, 3.1-30.2). Patients were divided into high and low SUVmax groups according to the median SUVmax. Representative examples of patients with surgical pulmonary SCC who had a high or low SUVmax are shown in Figure 1. The demographic and clinical characteristics of the study population are shown in Table 1.

**Table 1:** Patient characteristics

Characteristic	No. of patients
**Age**	
≤65	56
>65	28
**Gender**	
Male	65
Female	19
**Smoking status**	
Smoker	61
Non-smoker	23
**ECOG**	
0	30
1-2	54
**Differentiation**	
Well	27
Moderate/poor	57
**Tumour size**	
≤3cm	47
>3cm	37
**Lymph node metastasis**	
Negative	45
Positive	39
**Pathologic stage**	
IA	16
IB	14
IIA	13
IIB	20
IIIA	18
IIIB	3
**Adjuvant therapy**	
Adjuvant chemotherapy	54
Adjuvant chemoradiotherapy	12
**Surgical procedure**	
Lobectomy	67
Wedge resection	4
Sleeve lobectomy	9
Pneumonectomy	4
Tumor location	
Right upper lobe	28
Right middle lobe	9
Right lower lobe	13
Left upper lobe	23
Left lower lobe	11

### PD-L1 protein expression

Positive PD-L1 immunostaining was observed in the membranes and/or cytoplasms of tumour cells. Representative examples of PD-L1 staining patterns are shown in Figure 1. Positive PD-L1 protein expression was noted in 49 of the 84 patients with SCC enrolled in this study (58.3%).

### Correlations between PD-L1 expression and the SUVmax and clinicopathological characteristics

The correlations between PD-L1 expression and the SUVmax and clinicopathological characteristics are presented in Table 2. PD-L1 positivity was noted more frequent in patients with moderately or poorly differentiated disease than in patients with well-differentiated disease (P=0.006). However, there were no significant correlations between PD-L1 expression levels and age, gender, smoking history, ECOG, tumour size, lymph node metastasis, TNM stage, serum CEA levels, serum SCC levels or serum CYFRA 21-1 levels. A high SUVmax was significantly correlated with histological differentiation (P=0.037) and lymph node metastasis (P=0.025). No correlations were noted between a high SUVmax and age, gender, smoking history, ECOG, tumour size, TNM stage, serum CEA levels, serum SCC levels or serum CYFRA 21-1 levels. Spearman's analysis showed that PD-L1 expression was associated with a higher SUVmax (Table 3) (*rho* = 0.23; P = 0.035).

**Table 2:** Associations between clinicopathologic parameters and PD-L1 expression and the SUVmax

Clinicopathologic characteristics	All patients n(%)	PD-L1		*P-value*	SUVmax		*P*-value
		Negative	Positive		Low	High	
**Age**				0.118			0.164
≤65	56	20	36		23	33	
> 65	28	15	13		16	12	
**Gender**				0.103			0.538
Male	65	24	41		29	36	
Female	19	11	8		10	9	
**Smoking history**				0.23			0.103
Smoker	61	23	38		25	36	
Non-smoker	23	12	11		14	9	
**ECOG**				0.248			0.344
0	30	15	15		16	14	
1-2	54	20	34		23	31	
**Differentiation**				0.006			0.037
Well	27	17	10		17	10	
Moderate/poor	57	18	39		22	35	
**Tumor size**				0.281			0.161
≤3 cm	47	22	25		25	22	
> 3 cm	37	13	24		14	23	
**Lymph node metastasis**				0.149			0.025
Negative	45	22	23		26	19	
Positive	39	13	26		13	26	
**TNM stage**				0.248			0.161
I	30	15	15		17	13	
II-III	54	20	34		22	32	
**Serum CEA levels**				0.488			0.509
Negative	64	28	36		31	33	
Positive	20	7	13		8	12	
**Serum SCC levels**				0.151			0.257
Negative	55	26	29		28	27	
Positive	29	9	20		11	18	
**Serum CYFRA 21-1 levels**				0.18			0.101
Negative	48	23	25		26	22	
Positive	36	12	24		13	23	

Serum marker standard cut-off, CEA=5.0ng/ml; CYFRA 21-1=3.3 ng/ml; SCC=15.2 ng/ml.

**Table 3:** Correlation between PD-L1 expression and the SUVmax

PD-L1	n	SUVmax		*rho*	*P*
		High	Low		
Positive	49	31	18		
Negative	45	24	21	0.23	0.035

### Prognostic value of PD-L1 expression and the SUV max

**Figure 2:**
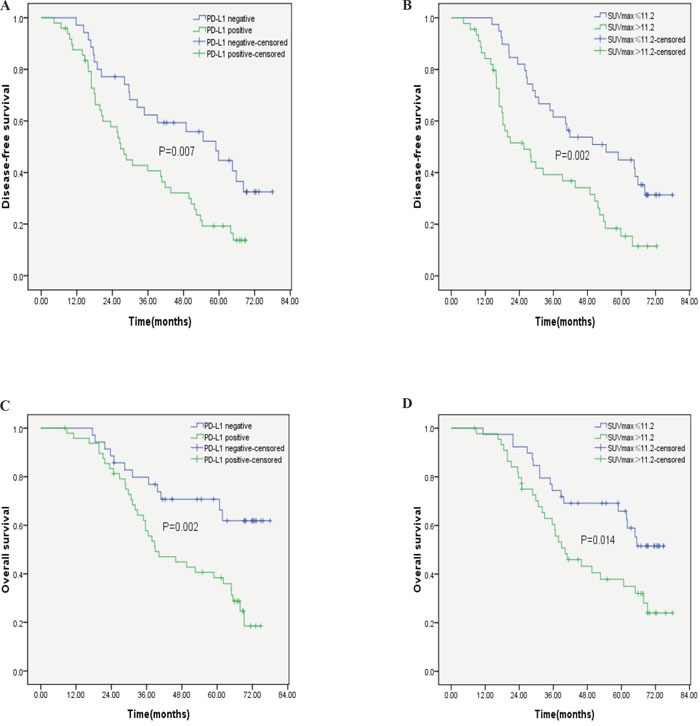
Kaplan-Meier survival curves for patients with pulmonary SCC **(A)** DFS curves for patients with negative PD-L1 expression and patients with positive PD-L1 expression. **(B)** DFS curves for patients with a low SUVmax and patients with a high SUVmax. **(C)** OS curves for patients with negative PD-L1 expression and patients with positive PD-L1 expression. **(D)** OS curves for patients with a low SUVmax and patients with a high SUVmax.

PD-L1-positive patients displayed significantly shorter DFS (P=0.007) and OS (P=0.002) than PD-L1-negative patients. Furthermore, patients with a high SUVmax displayed shorter DFS (P=0.002) and OS (P=0.014) than patients with a low SUVmax.(Figure 2). The univariate Cox regression model showed that tumour differentiation, lymph node metastasis, TNM stage, PD-L1 expression and the SUVmax were correlated with OS, whereas age, gender, smoking history, tumour size, serum CEA levels, serum SCC levels, and serum CYFRA21-1 levels were not significantly correlated with OS. Additional multivariate analyses demonstrated that tumour differentiation, lymph node metastasis, TNM stage, PD-L1 expression and the SUVmax were significant independent predictors of OS (Table 4).

**Table 4:** Univariate and multivariate analyses of prognostic factors for overall survival

Factor	Univariate analysis		Multivariate analysis	
	HR (95%CI)	*P-*value	HR (95%CI)	*P*-value
Age(>65 vs ≤65)	1.212 (0.668–2.199)	0.527		
Gender (male vs female)	1.275 (0.633–2.565)	0.496		
Smoking history (yes vs no)	1.149 (0.606-2.179)	0.670		
Tumour differentiation (poor vs moderate/good) *	3.545 (1.687-7.451)	0.001	4.909(2.149-11.218)	<0.001
Tumor size(>3cm vs ≤3cm)	1.621 (0.913–2.881)	0.099		
Lymph node metastasis (yes vs no) *	2.366 (1.323-4.230)	0.004	2.504(1.343-4.671)	0.004
TNM stage (III -II vs I) *	3.427 (1.727-6.798)	<0.001	3.17(1.550-6.483)	0.002
Serum CEA levels(>5 ng/ml vs ≤5 ng/ml)	1.284 (0.653–2.525)	0.468		
Serum SCC levels(>15.2 ng/ml vs ≤15.2 ng/ml)	1.678 (0.943-2.987)	0.079		
Serum CYFRA 21-1 levels(>3.3 ng/ml vs ≤3.3 ng/ml)	1.479 (0.834-2.625)	0.181		
PD-L1 (positive vs negative) *	2.667 (1.378–5.164)	0.004	2.489(1.271-4.876)	0.008
SUVmax (high vs low) *	2.085 (1.148-3.787)	0.016	2.007(1.086-3.709)	0.026

*P<0.05.

## DISCUSSION

PD-L1 is a novel member of B7/CD28 superfamily and is more highly expressed in tumour tissues than in normal tissues [5]. In our previous study, we found that PD-L1 over expression was associated with significantly shorter OS in gastric cancer and breast cancer [26–27]. However, the clinical significance of PD-L1 expression in surgically resected SCC specimens has not been fully characterized. We therefore investigated PD-L1 expression and evaluated its prognostic significance in patients with SCC. In our study, positive PD-L1 staining was observed in 58.3% of patients. This rate of positive PD-L1 expression noted in our study was similar to that noted in a previous study on SCC, in which positive PD-L1 expression was detected in 56.2% of patients with SCC [28]. In addition, our results demonstrated that increased PD-L1 expression was significantly associated with poor survival in patients with SCC, a result consistent with those of a previous study [29]. These results support the idea that increased PD-L1 expression enables tumour cells to evade host immune surveillance and promotes disease progression [30]. In contrast, the results of another previous study showed that high PD-L1 expression levels were significantly associated with a favourable prognosis in SCC [28]. The study of Taso et al. [31] demonstrated that positive PD-L1 expression was not correlated with OS in resected NSCLC. Increased PD-L1 expression has been shown to be correlated with favourable and unfavourable prognoses in different studies. These findings may be attributable to the use of different thresholds to determine PD-L1 positivity, as well as heterogeneity with respect to baseline clinical and pathological features. Future studies aiming to determine the clinical applicability of PD-L1 must endeavor to develop a standardized protocol with which PD-L1 expression can be assessed.

The SUVmax is the 18F-FDG PET/CT parameter most commonly used for making diagnoses, performing TNM staging and monitoring for therapeutic effects because of its high reproducibility and availability. In addition, various studies have described the prognostic significance of SUVmax in both early and advanced NSCLC [32–33]. A systematic review and meta-analysis showed that a high SUVmax is related to inferior overall survival in patients with NSCLC [34]. However, the prognostic significance of the SUVmax in SCC of the lung remains controversial. The goal of this study was to evaluate the significance of the SUVmax as a prognostic factor in SCC. We found that a high primary tumour SUVmax is a poor prognostic factor in patients with SCC. In contrast, Tsutani and colleagues showed that a high primary tumour SUVmax was a powerful prognostic determinant in patients with ADC, but not in patients with SCC of the lung [35]. The use of different SUVmax cut-offs, as well as differences in sample sizes, may account for the differences in outcomes between our study and that mentioned above. We also investigated the biological characteristics of tumours with high and low FDG uptake. Our study showed that the SUVmax was strongly correlated with lymph node metastasis and tumour differentiation in patients with SCC.

A novel therapy based on PD-1/PD-L1 inhibitors known to have impressive antitumour activity in patients with NSCLC recently became the standard therapy for NSCLC [36]. In this age of personalized medicine, clinicians are faced with the following important question: how can physicians identify patients who are more likely to benefit from anti-PD-1/PD-L1 treatment? The findings of recent studies indicate that increased PD-L1 expression has emerged as a predictive biomarker useful for stratifying patients with NSCLC who are receiving PD-1/PD-L1 therapeutic agents [37]. In the present study, we investigated the relationship between PD-L1 expression and clinicopathological factors. Our results showed that PD-L1 positivity was more frequent in patients with moderately or poorly differentiated disease than in patients with well-differentiated disease, suggesting that PD-L1 expression can serve as a marker of disease progression. These patients may benefit more from PD-1/PD-L1 immune checkpoints inhibitors than other patients. In addition, several studies have described the feasibility of performing immuno-PET imaging with radiolabelled PD-1 and PD-L1 antibodies in murine models [38–39]. The present study investigated whether the SUVmax can predict tumour tissue PD-L1 expression levels in patients with SCC. We observed an association between SUVmax and PD-L1 expression. Our findings suggest that FDG-PET may serve as a noninvasive tool for assessing PD-L1 expression and can therefore predict the benefits that may be derived from PD-1/PD-L1 pathway-targeted immunotherapy.

The present study had several limitations. First, the samples and data described herein were collected retrospectively, which may have led to bias resulting from variations among the treatment protocols used to manage the patients in question. Second, the limited sample size of the study cohort may have reduced the statistical power of the data analysis. Third, specific cut-off values for PD-L1 positivity have not been clearly defined, and the reproducibility of results associated with specific PD-L1 cut-off values has not been formally assessed. Fourth, different studies used different antibodies, and the choice of antibody may influenced the results of studies. In the present study, we used the clone 28-8 antibody, which has been used in clinical trials regarding nivolumab. Different anti-PD-L1 antibodies may need to be validated in the same SCC sample in future studies. Fifth, the patients included in our study had stage I-III disease; therefore, it is unclear whether our results are applicable to patients with stage IV disease. A prospective study with a larger sample size is warranted to validate our findings.

In conclusion, our results suggested that high PD-L1 expression levels and a high SUVmax were associated with a poor prognosis in surgical pulmonary SCC. The existence of a significant correlation between the SUVmax and PD-L1 expression levels serves as justification for exploring the usefulness of the SUVmax as a predictor of PD-1/PD-L1 inhibitor activity.

## MATERIALS AND METHODS

### Patients and samples

Ninety-five patients with known diagnoses of SCC underwent whole-body ^18^F-FDG PET/CT for initial disease staging before undergoing surgical resection with systematic lymph node dissection at Harbin Medical University Cancer Hospital from January 2010 to December 2013. Archival materials including patient demographics, clinical data and follow-up information were available for all patients. Data on patient demographics, clinicopathological features and overall survival were extracted from medical records. Five patients who received preoperative chemotherapy or radiotherapy were excluded from the study, as were six patients who were examined at our PET/CT center more than 4 weeks before undergoing surgical treatment. Thus, 84 patients were included in the study. Tumour stage was determined according to the seventh edition of the AJCC system, which was published in 2009. The study was approved by the Ethics Committee of Harbin Medical University Cancer Hospital.

### PET/CT protocol and imaging analysis

The FDG-PET protocol used in this study was described in our previous report [40]. In this study, the output results, including the SUVmax, were assessed by two imaging physicians with experience with PET/CT imaging and familiarity with PET-VCAR software and our PACS system. All images were reviewed to localize the target lesions identified by the above two imaging physicians, and any discrepancies regarding the images were resolved by discussion and the achievement of consensus.

### Immunohistochemistry (IHC)

IHC was performed using standard indirect immunoperoxidase procedures. Briefly, 4μm-thick sections from a paraffin-embedded tissue block were de-waxed with xylene and rehydrated with a graded ethanol series and distilled water. Endogenous peroxidase activity was subsequently blocked using 3.0% hydrogen peroxide for 10 minutes, after which the tissue sections were incubated with the indicated primary antibodies (anti-PD-L1 antibody, clone 28-8, Abcam, Cambridge, UK) for 60 minutes at room temperature. For antigen visualization, we immersed the sectionsin 3, 3-diaminobenzidine solution and counterstained them with hematoxylin.

The PD-L1 immunostaining results were classified into two groups based on staining intensity and tumour cell positivity. Staining intensity was scored as follows: 0, negative staining; 1, weak staining; 2, moderate staining; and 3, strong staining. All cases in which more than 5% of tumour cells displayed a staining intensity ≥2 were considered positive. Cases with staining intensity <2 or less than 5% of tumour cells were considered negative. The 5% threshold used herein was chosen based on the results of a previous clinical trial [41–42].

### Statistical analysis

The correlations between PD-L1 expression and clinicopathological characteristics were evaluated using chi-square tests or Fisher's exact test. Spearman’ s correlation coefficient (rho) was used for rank correlation. Disease-free survival and overall survival were calculated using by the Kaplan–Meier method, and comparisons were performed using the log-rank test. Univariate and multivariate regression was performed using the Cox proportional hazards model. Two-sided *p* values <0.05 were considered statistically significant. All statistical analyses were performed using SPSS software (version 17.0; SPSS, Chicago, Illinois, USA).
